# Supplementary material for: Phylogenetic and Expression Analysis of Fos Transcription Factors in Zebrafish
Source: Int J Mol Sci. 2022 Sep 3;23(17):10098. doi: 10.3390/ijms231710098 (PMC9456341; doi:10.3390/ijms231710098)
Supplement: Supplementary file 1 [file ijms-23-10098-s001.zip › ijms-1901036-supplementary.pdf]

Phylogenetic and Expression Analysis of Fos Transcription Factors in Zebrafish

Khadizatul Kubra [1], Gurveer K. Gaddu [1], Clifford Liongue [1,2], Somayyeh Heidary [1], Alister C. Ward [1,2], Amardeep S. Dhillon [1,2,3,4,*] and Faiza Basheer [1,2,*]

[1] School of Medicine, Deakin University, Geelong, VIC 3216, Australia
[2] Institute of Mental and Physical Health and Clinical Translation, Deakin University, Geelong, VIC 3216, Australia
[3] Olivia Newton-John Cancer Research Institute, Melbourne, VIC 3084, Australia
[4] School of Cancer Medicine, LaTrobe University, Melbourne, VIC 3086, Australia
* Correspondence: amardeep.dhillon@deakin.edu.au (A.S.D.); faiza.basheer@deakin.edu.au (F.B.)

Supplementary data

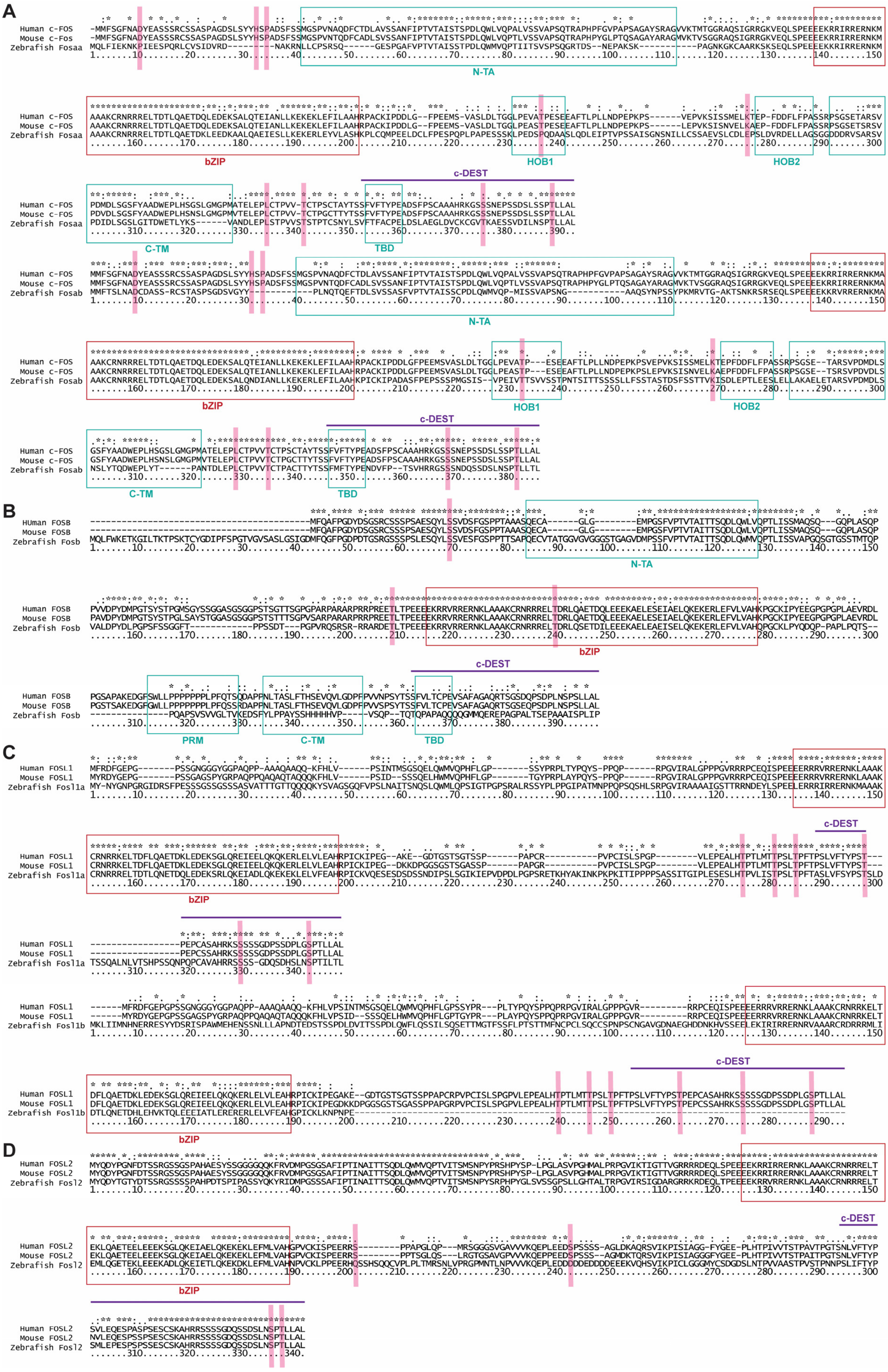

A
Human c-FOS
Mouse c-FOS
Zebrafish Fosaa
Zebrafish Fosab
N-TA
bZIP
HOB1
HOB2
c-DEST
C-TM
TBD
B
HUman FOSB
Mouse FOSB
Zebrafish Fosb
PRM
C
Human FOSL1
Mouse FOSL1
Zebrafish Fosl1a
Zebrafish Fosl1b
D
Human FOSL2
Mouse FOSL2
Zebrafish Fosl2

Figure S1. Multiple sequence alignment of mammalian and zebrafish proteins. Alignment of amino acid sequences of human, mouse and zebrafish FOS (A), FOSB (B), FOSL1 (C) and FOSL2 (D) proteins. Boxes and solid lines indicate key functional domains including N-TA, HOB1, HOB2, PRM, C-TM, TBD (blue box), bZIP domain (red box) and C-DEST domain (purple solid line). Pink solid lines on the sequences indicate previously identified regulatory phosphorylation sites.

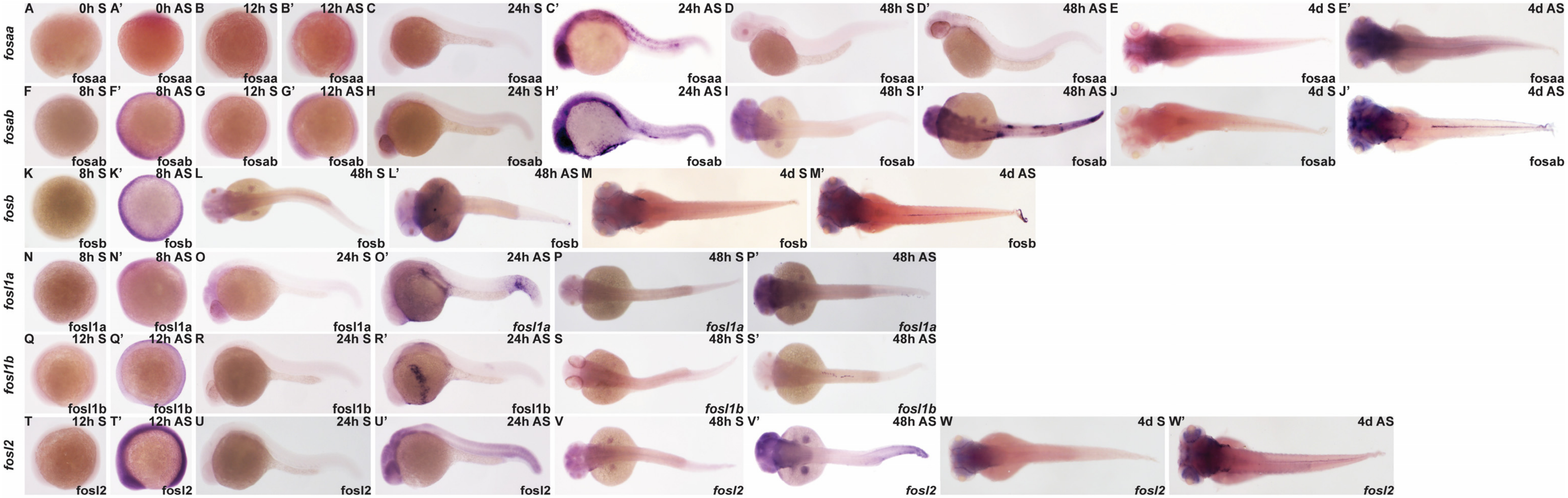

Figure S2: Spatio-temporal expression of control probes (S) vs their respective specific gene expression probes (AS) during early and late zebrafish embryonic development. Lateral view of sense and anti-sense stained embryos showing expression for *fosaa* at 0 hpf (A-A'), 12 hpf (B-B'), 24 hpf (C-C'),48 hpf (D-D') and 4 dpf (E-E'); *fosab* at 8 hpf (F-F'), 12 hpf (G-G'), 24 hpf (H-H'), 48 hpf (I-I') and 4 dpf (J-J'); *fosb* at 8 hpf (K-K'), 48 hpf (L-L') and 4 dpf (M-M'); *fosl1a* at 8 hpf (N-N'), 24 hpf (O-O') and 48 hpf (P-P'); *fosl1b* at 12 hpf (Q-Q'), 24 hpf (R-R') and 48 hpf (S-S'); and *fosl2* at 12 hpf (T-T'), 24 hpf (U-U'), 48 hpf (V-V') and 4 dpf (W-W').

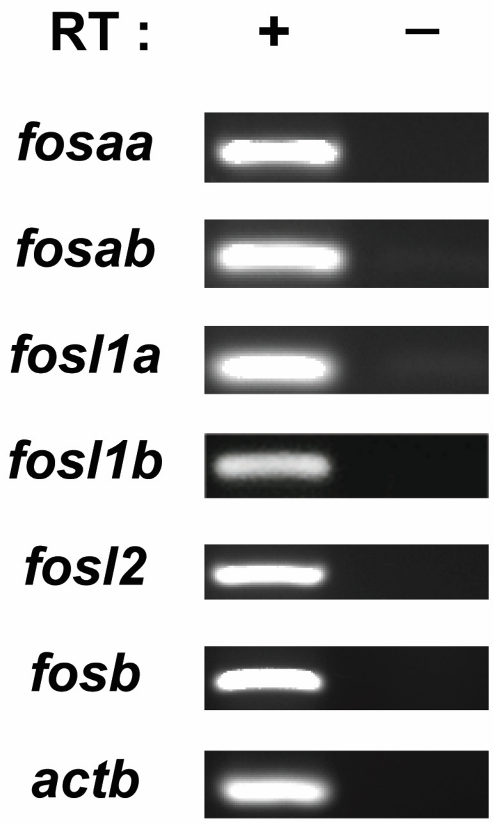

Figure S3: Control RT-PCR reaction analysis of zebrafish *fos* genes by RT-PCR using total RNA from 5 dpf embryos with (+) and without (-) RT.

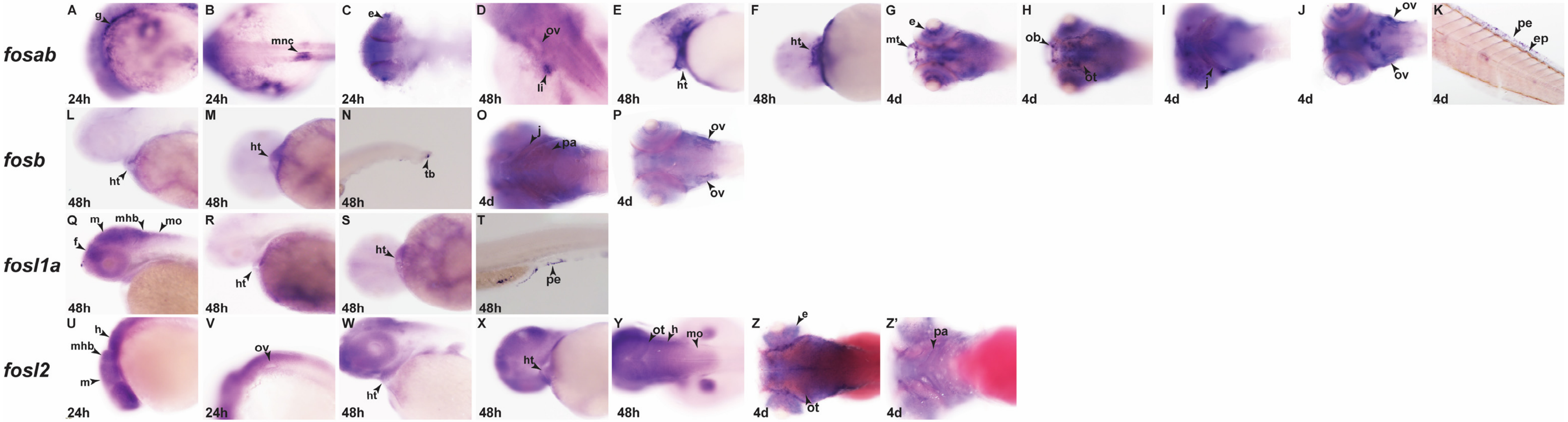

Figure S4: Magnified spatio-temporal expression of *fos* genes in specific organs or cell lineage of zebrafish embryos. Lateral, dorsal or frontal views of zebrafish embryos analysed by WISH showing expression of *fosab* at 24 hpf (A-C), 48 hpf (D-F) and 4 dpf (G-K); *fosb* at 48 hpf (L-N) and 4 dpf (O-P); *fosl1a* at 48 hpf (Q-T); and *fosl2* at 24 hpf (U-V), 48 hpf (W-Y) and 4 dpf (Z-Z'). e, eye; ep, epidermis; f, forebrain; g, gut; ht, heart; j, jaw; li, liver; m, midbrain; mhb, midbrain hindbrain barrier; mnc, migratory neural crest; mo, medulla obolongata; mt, mouth; ob, olfactory bulb; ot, optic tectum; ov, otic vesicle; pa, pharangeal arch; pe, peridermis; tb, tailbud.
